# Rumination and Activity Patterns in Angus and Angus-Cross Beef Calves: Influences of Sex, Breed, and Backgrounding Diet

**DOI:** 10.3390/ani12141835

**Published:** 2022-07-19

**Authors:** Bobwealth Omontese, Friday Zakari, Megan Webb

**Affiliations:** 1 Department of Food and Animal Sciences, Alabama A & M University, Normal, AL 35762, USA; 2 Department of Veterinary Physiology, Biochemistry and Pharmacology, Faculty of Veterinary Medicine, University of Jos, Jos PMB 2084, Nigeria; fridayzakari@gmail.com; 3 Community Engagement & Partnerships, Eastern West Virginia Community and Technical College, Moorefield, WV 26836, USA; megan.webb@easternwv.edu

**Keywords:** circadian rhythm, activity behavior, beef calves, weaned calves

## Abstract

**Simple Summary:**

Rumination and activity behavior are important indices for monitoring the welfare and health status of beef cattle. Stress, excitement, and diseases can alter the rumination and activity patterns of beef cattle. Backgrounding allows producers to feed a variety of forages to improve growth performance before the beef calves enter the feedlot. This study was designed to evaluate the influences of sex, breed, and backgrounding diet on rumination, and the activity patterns in Angus and Angus-cross beef calves. Moreover, the daily variations in rumination and activity in Angus and Angus-cross beef calves under different backgrounding systems were studied. Our results demonstrated that the time of the day and backgrounding diet influenced rumination and activity patterns of Angus and Angus-cross beef calves.

**Abstract:**

The objectives of this study were to evaluate the influences of sex, breed, and backgrounding diet on rumination and activity patterns in Angus and Angus-cross beef calves; and the daily variations in rumination and activity in Angus and Angus-cross beef calves under different backgrounding systems. A total of 62 freshly weaned calves were vaccinated and randomly stratified by sex (heifers and steers), breed (Angus and Angus × Simmental cross), and assigned randomly to 3 backgrounding treatments for 55 days. The peak values for rumination and activity in heifers, steers, Angus, and Angus × Simmental cross occurred during the dark and light phases of the dark/light cycle, respectively. Beef calves backgrounded on cover crops had higher (*p* < 0.05) rumination (45.33 ± 1.57 min) compared with calves backgrounded on a perennial pasture (43.96 ± 1.47 min) diet. Similarly, drylot calves (24.16 ± 0.68 min) had higher (*p* < 0.05) activity compared to perennial pasture (23.49 ± 0.72 min). The results showed that sex and breed did not influence rumination and activity of Angus and Angus-cross beef calves during the study period. We concluded that the time of the day and backgrounding diet influenced rumination and activity patterns of Angus and Angus-cross beef calves.

## 1. Introduction

Rumination is a natural behavioral process in ruminants, which involves regurgitation of previously consumed feed and masticating it a second time [1]. Diet composition plays a major factor in determining the frequency of rumination, with increased forage intakes resulting in increased frequency of rumination [2]. Rumination has long been associated with health in cattle [3] and, more recently, changes in rumination have been used to assess the responses of cattle to acute stressors [4] and disease [5]. Measuring rumination behavior in calves is of great importance for monitoring rumen development, evaluating diets, and roughage intake, as well as calf health and well-being [6,7,8,9]. The general welfare and metabolic conditions of ruminants are often assessed by the examination of rumen functioning [10,11]. Monitoring and tracking rumination activity may provide essential information, mainly including changes in feeding quality, voluntary feed intake, grazing quality and quantity, metabolic processes, weather influence, and the presence of diseases influencing the appetite of cattle [12]. Changes in rumination activity are the earliest warning signs informing the veterinarian about potential health problems [13]. Continuous monitoring of behavioral and physiological parameters can aid in the early detection of sick animals, allowing immediate and targeted therapy [14,15]. In livestock, data on activity and rumination have been used to measure the effects of different rations on ruminal functions [16]. Differences in rumination activity have been reported between breeds [17] as well as body sizes [18], demonstrating that cattle with high feed intake have shorter times for ruminating and chewing [13]. Previous work on lying behavior, activity, and rumination in cattle has focused mostly on mature cattle [19,20,21,22,23,24].

Backgrounding is the practice of rearing a calf on a combination of forage, such as pasture and grains, in order to increase its weight before it is placed in a feedlot. It is the intermediate stage between when the calf is weaned and when it is placed in a feedlot [25]. The main aim of backgrounding is to maximize weight gain primarily in the form of muscle and frame development, with little fat deposition during the growing phase [26,27]. Klinger et al. [28] observed that backgrounding affects feed efficiency, nutrient digestibility, activity behavior, and rumination in crossbred steers. It plays an important role in minimizing health issues once in the feedlot or intensive finishing system by allowing livestock to interact, experience low levels of contagions, and develop immunity.

Circadian rhythms are physiologic processes with a cyclical periodicity of approximately 24 h, generated by the endogenous biological pacemaker, the suprachiasmatic nucleus found in the anterior hypothalamus [29]. These rhythms regulate a variety of biological processes, such as body temperature, feeding, and hormone secretion. The circadian rhythm of activity is a variable that has high potential to be a key indicator for the general physiologic state of animals, as its rhythmicity is an outcome of a large number of physiological processes [30]. Moreover, strong rhythmicity of activity is known to be a characteristic of healthy and adapted organisms [31,32]. Exposure to stressors may affect or disrupt the physiological rhythm in animals. As a result, the internal synchronization may be influenced, leading to chronic difficulties for the health and well-being of an organism. The present study was designed to obtain more insight into the temporal dynamics of the circadian rhythm of rumination and activity behavior in beef calves and to study how the stressor may affect rhythmicity.

In summary, the objectives of the study were to evaluate (1) the influences of sex, breed, and backgrounding diet on rumination and activity patterns in Angus and Angus-cross beef calves; and (2) the daily variations in rumination and activity in Angus and Angus-cross beef calves under different backgrounding systems. We hypothesized that rumination and activity patterns would differ according to time of day, calf sex, breed, and backgrounding diet.

## 2. Materials and Methods

### 2.1. Animals and Preweaning Management

This study was conducted at the University of Minnesota North Central Outreach Research Station (NCROC), Grand Rapids, MN. Angus and Angus x Simmental crossbred calves were used, born within a 23-d period with an average birth weight of 35.5 kg, from cows with an average age of 4.7 years (range 2–13). Birth weights for the Angus and Angus-cross were 36.09 and 34.54 kg, respectively. Weight variabilities for the Angus and Angus-cross were 13.82% and 15.40%, respectively. Cow–calf pairs grazed from a mix of introduced pasture grasses typical of a Northern Mixed Prairie throughout the preweaning period. Male calves were castrated between 2.8 and 3.6 months of age. All calves were fence-line weaned for 6 d prior to enrollment in the study. At weaning, all calves were weighed (BW) in a hydraulic squeeze chute (Tru-Test XR 3000, Mineral Wells, TX, USA) with load cells mounted under the chute, vaccinated using a bacterin-toxoid against clostridial diseases (Ultra Choice 8, Zoetis, Parsippany, NJ, USA) and a modified live vaccine for the prevention of respiratory viruses and Mannheimia haemolytica (Titanium 5 + PH-M, Elanco Animal Health, Greenfield, IN, United States of America). Calves were dewormed (Valbezene, Zoetis, Parsippany, NJ, USA) and treated with Cydectin (Bayer, Shawnee Mission, KS, USA) for control of ectoparasites.

### 2.2. Experimental Design

A total of 62 (n = 62) freshly weaned calves were randomly stratified by sex (heifers (n = 30) and steers (n = 32)), breed (Angus (n = 40), and Angus × Simmental cross (n = 22)). The 62 (n = 62) freshly weaned calves were vaccinated and randomly stratified by sex (heifers and steers) and breed (Angus and Angus × Simmental cross) and assigned randomly to 1 of 3 backgrounding treatments for 55 d: (1) a high roughage diet delivered in a bunk within a feedlot (drylot; DL); (2) perennial pasture vegetation within rotational paddocks (perennial pasture; PP); or (3) summer annual cover crop within a strip plot (cover crop; CC). On d 56, calves were commonly placed into the feedlot located at the same premise and delivered the same high-energy diet for 70 days until harvest. All experimental animals received a free-choice mineral (Wind and Rain, Purina Animal Nutrition LLC, MN) throughout the 55-d backgrounding period. 

### 2.3. Backgrounding Management and Dietary Treatments

Drylot backgrounded calves were fed a high roughage haylage-based ration delivered every morning (0800 h) using a truck-mounted (F-Series, Ford Motor Company, 120 Dearborn, MI, USA) total-mixed ration mixer (KUHN KNIGHT Model Auggie 3136, Brodhead, WI, USA) and fitted with a scale with 4.5 kg of resolution (Weigh-Tronix, Fairmont, MN, USA). The diet was formulated by Purina Animal Nutrition, LLC, to contain 1.06 Mcal NEg/kg, 12.5% CP, and vitamins and minerals to meet requirements for growing cattle [33]. Moreover, the DL diet was formulated to deliver 230 mg of monensin per head daily. The paddocks used in this experiment containing perennial vegetation have historically been managed as rotational pastures. Forage species within PP consisted of a mix of perennial ryegrass (*Lolium perenne*), quackgrass (*Elymus repens* (L.) Gould), orchardgrass (*Dactylis glomerata* (L.)), smooth bromegrass (*Bromus inermis* (L.)), red clover (*Trifolium pratense* (L.)), and alfalfa (*Medicago sativa* (L.)) in various proportions. Each pasture paddock used in the experiment was approximately 2.43 ha. Each pasture paddock was sequentially sampled for total available forage prior to grazing. Stocking rates were then set by calculating an estimated forage allowance at 70% utilization. Calves grazing PP were then rotated between equally divided multiple pastures to ensure adequate estimated forage allowance.

The summer annual CC was seeded using a Great Plains no-till drill (Great Plains, Salina, KS, USA) 60 d (21 July 2018) before the commencement of the study. In addition, nitrogen (N) fertilizer within the acceptable range (120–140 pounds per acre, Minnesota Department of Agriculture) was applied at the time of CC seeding. Annual CC was allowed to grow with no additional treatment or defoliation until the initiation of this study. Annual CC vegetation consisted of 82% cereal oats (Avena sativa var. Mustang), 7.6% purple top turnips (Brassica rapa subspp. Rapa var. Purple Top), 7.6% Hunter forage brassica (Brassica spp. Var. Brassica rapa subspp. Pekinensis × Brassica rapa subspp. Rapa), and 2.6% Graza forage radish (Raphanus raphanistrum subspp. Sativus var. Graza). The total seeding rate was 44.32 kg ha^−1^. Prior to grazing, the square-shaped study area was divided in half using a high-tensile electric fence to allow for strip grazing of the study area. Each strip was established at a pre-set size of 10% of the total study area. Each strip was sequentially sampled weekly for the total available forage. Stocking rates were then set by calculating an estimated forage allowance at 70% utilization. Calves grazing CC were permitted access to forward strips according to the estimated forage allowance. Cattle had ad libitum access to water and minerals (Wind and Rain, Purina Animal Nutrition LLC, MN) throughout the 55-d backgrounding period. Nutrient composition is the backgrounding diet, which can be found below (Table 1). 

### 2.4. Feedlot Management

On d 56 (end of backgrounding), calves were vaccinated using a bacterin-toxoid for clostridial diseases (Ultra Choice 8, Zoetis, Parsippany, NJ, USA) and a modified live vaccine for the prevention of respiratory viruses and Mannheimia haemolytica (Titanium 5 + PH-M, Elanco Animal Health, Greenfield, IN, USA). All calves were dewormed (Valbezene, Zoetis, NJ, USA), and treated with Cydectin (Bayer, Shawnee Mission, KS, USA) for control of ectoparasites. Cattle grazing PP and CC were transported 3.38 km to the North farm feedyard, NCROC, the University of Minnesota, where they were separated by gender into 2 different pens and acclimated to the bunk for 14 d prior to finishing. Steers and heifers were rotated between pens every 28 d to minimize the pen effect.

### 2.5. Evaluation of Activity Behavior and Rumination in Beef Calves

After the termination of backgrounding, the durations of rumination and activity were monitored bihourly for 48 h from 0:00 h to 48:00 h (GMT + 1) using an ear tag activity monitor (SCR eSense, Allflex, Irving, TX, USA). The average value of the measured data for 2 consecutive days was obtained to have final readings from 0:00 h to 24:00 h. The rumination and activity were recorded three times with an interval of one week during the study period. Calves had one week to habituate to the ear tag and the study settings before the study started. For reliable identification during observations, calves were marked with individual numbers ranging from 1 to 62 on each side of the croup. Two people were needed to fit the tags on the calves. When assembling the tags, the tag number and cow number were recorded. The Allflex eSense™ ear tag sensors were positioned in the middle of the calves’ left ears, which recorded rumination and activity behavior. The activity and rumination data were recorded by the sensors in minutes per 2 h intervals as arbitrary numbers determined by a three-dimensional accelerometer that recorded the speed and angle of the ear movements. Data were transmitted from the tag to the antennae in the office every 20 min where the system software reported changes in activity and rumination using a series of internal algorithms, which were proprietary to SCR (SCR eSense, Allflex, Irving, TX, USA). Activity behavior represents the sum of a range of different behaviors, including eating/grazing, drinking, locomotion, and lying [34]. 

### 2.6. Measurement of Environmental Parameters

Ambient temperature, relative humidity, and wind speed during the study period were collected from a meteorological station located within the NCROC and 224 Itasca County Airport-Gordon Newstrom Field in Grand Rapids, MN, USA.

### 2.7. Statistical Analysis

Data obtained were expressed as mean ± standard error of the mean (mean ± SEM). Rumination and activity behavior values were found to be normally distributed as determined by the D’Agostino and Pearson Omnibus normality test. A nonlinear regression analysis was used to determine the daily rhythm in rumination and activity of the calves. An independent *t*-test was used to determine the effects of sex and breeds on rumination and activity. One-way analysis of variance (ANOVA) followed by Tukey’s post hoc test was used to compare the effect of background diet on rumination and activity. The diurnality index was computed by dividing the number of activity counts during the light phase of the light/dark cycle by the total number of activity counts during the whole day (photophase and scotophase) for each individual. A scale from 0 to 1 was used to classify the diurnality index. An index of 0.5 indicates that the behavior was performed equally as often during the photophase and the scotophase of the light–dark cycle. An index above 0.5 or close to 1 indicates that the behavior was mostly or only performed during the photophase period of the light–dark cycle. An index of 0 or below 0.5 means that the behavior was performed only or mostly during the scotophase [35]. The relationship between rumination and activity behavior was analyzed using Pearson’s correlation. Data were analyzed using GraphPad Prism software, version 6.01, for Windows (GraphPad Software, San Diego, CA, USA) (www.graphpad.com (accessed on 05 March 2022)). 

## 3. Results

### 3.1. Environmental Parameters during the Study Period

The mean ambient temperature, relative humidity, and wind velocity during the study period were −5.85 ± 1.41 °C, 72.14 ± 1.63%, and 7.63 ± 0.76 m/s, respectively. 

### 3.2. Effects of Sex, Breed, and Backgrounding System on Rumination and Activity in Beef Calves

The time spent on rumination and activity behavior was not affected (*p* > 0.05) by breed (Steer and heifer) or sex (Angus and Angus-Simmental cross) in beef calves. In contrast, beef calves backgrounded on cover crops (45.33 ± 1.57 min) spent more time (*p* < 0.04) ruminating compared to calves backgrounded on the perennial pasture (43.96 ± 1.47 min) diet. Calves backgrounded on drylot (24.16 ± 0.68 min) spent more time (*p* < 0.01) on activity behavior compared to those backgrounded on perennial pasture (23.49 ± 0.72 min) (Table 2).

### 3.3. Effects of Circadian Rhythm on Rumination and Activity Behavior in Beef Calves

**Figure 1:**
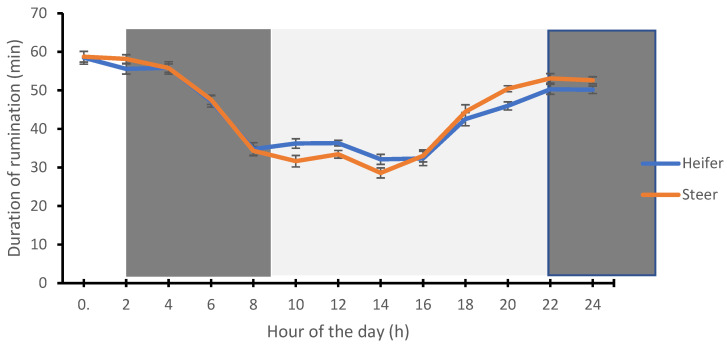
Circadian rhythms of rumination in heifer and steer calves. Each datum point represents the mean ± SEM of the heifer and steer at each measurement period. The light and dark shades represent the prevailing light–dark cycle.

**Figure 2:**
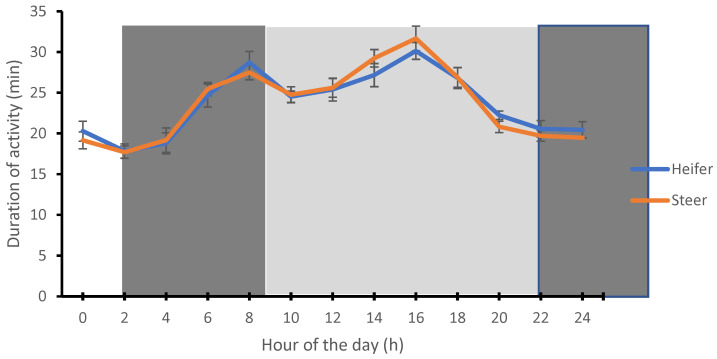
Circadian rhythms of activity behaviors in heifer and steer calves. Each datum point represents the mean ± SEM of the heifers and steers at each measurement period. The light and dark shades represent the prevailing light–dark cycle.

In the present study, the heifer (58.48 ± 1.66 min) and steer (58.74 ± 1.34 min) spent more time ruminating at midnight (0:00 h), while the lowest rumination time by the heifer (32.12 ± 1.30 min) and steer (28.59 ± 1.31 min) was recorded during the afternoon period (14:00 h) (Figure 1). The time spent on activity behavior by the heifer (30.14 ± 1.04 min) and steer (31.67 ± 01.52 min) were highest (*p* < 0.0001) during the afternoon period (16:00 h) and lowest (17.86 ± 0.88 min and 17.71 ± 0.76 min) during the night period (02:00 h) (Figure 2).

**Figure 3:**
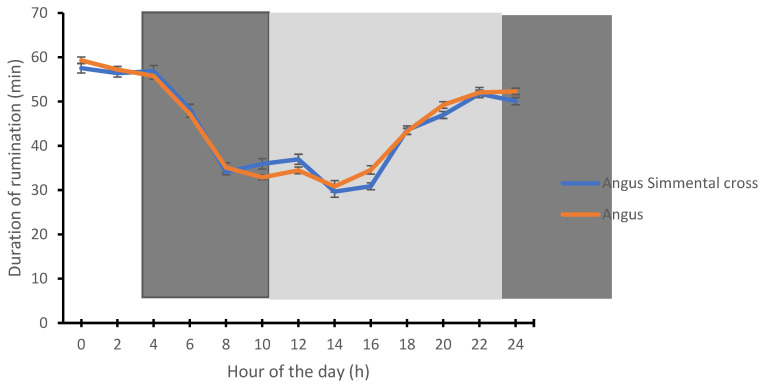
Circadian rhythms of rumination in Angus and Angus-cross calves. Each datum point represents the mean ± SEM at each measurement period. The light and dark shades represent the prevailing light–dark cycle.

**Figure 4:**
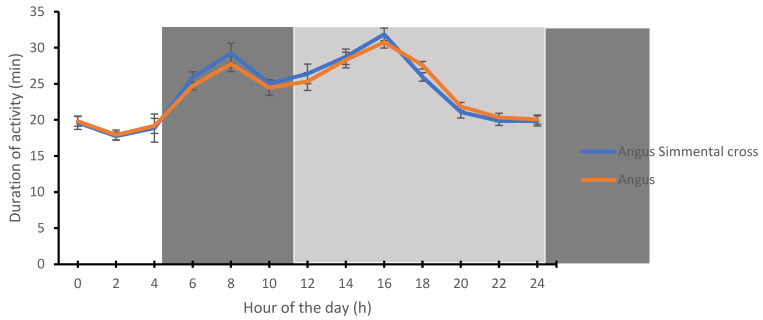
Circadian rhythms of activity in the Angus and Angus-cross. Each datum point represents the mean ± SEM of heifers and steers at each measurement period. The light and dark shades represent the prevailing light–dark cycle.

The rumination time was highest at midnight (0:00 h) for the Angus (59.31 ± 1.31 min) and Angus-cross (57.54 ± 1.28 min), respectively. In contrast, the lowest time spent on ruminating by the Angus (30.85 ± 0.75 min) and Angus-cross (29.66 ± 1.09 min) was recorded during the afternoon period (14: 00 h) (Figure 3). The time spent on activity by the Angus and Angus-cross was highest during the afternoon period (16:00 h) and lowest during the early morning hours (02:00 h) (Figure 4). 

### 3.4. The Diurnality Index of Activity Behavior and Rumination during the Study Period

**Figure 5:**
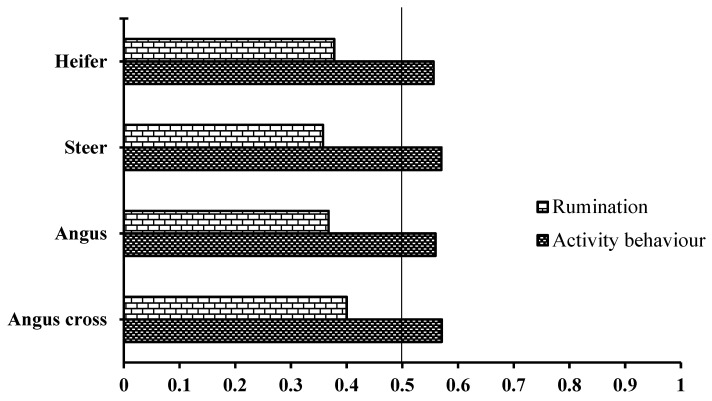
Diurnality index for rumination and activity behavior in the heifer, steer, Angus, and Angus-cross. The vertical line indicates the theoretical separation between nocturnal and diurnal activity (i.e., equal amounts of activity during the photophase and the scotophase of the light/dark cycle).

The diurnality index of activity behavior and rumination in the heifer, steer, Angus, and Angus-Simmental cross during the study period are shown in Figure 5. The diurnality indices of rumination for the heifer, steer, Angus, and Angus-Simmental cross were 0.3777, 0.3575, 0.3675, and 0.400, respectively. In contrast, the diurnality indices of activity behavior were 0.5561, 0.5700, 0.5596, and 0.5707 for the heifer, steer, Angus, and Angus-Simmental cross, respectively. 

### 3.5. Relationships between Rumination and Activity Behavior in Beef Calves

The correlation coefficient between rumination and activity behavior is presented in Table 3. There was a negative and significant (*p* < 0.0001) relationship between rumination and activity behavior in the heifer (r = −0.8517), steer (r = −0.8188), Angus (r = −0.8068), and Angus-Simmental cross (r = −0.8882).

### 3.6. Relationships between Environmental Parameters, Rumination, and Activity Behavior in Beef Calves

Correlation coefficients/relationship between rumination and activity behavior and the thermal environment parameters of ambient temperature, relative humidity, and wind velocity, recorded during the experimental period, are presented in Table 4.

## 4. Discussion

Application of the cosine model showed that rumination and activity behavior in beef calves exhibited a strong diurnal rhythm during the study period. Rumination values exhibited a gradual decrease and increase during the photophase and scotophase period of the light–dark cycle. The present results indicate that the frequency of rumination in beef calves peaked at night (0:00 h). This agrees with the findings by Beauchemin [2], Grant et al. [36], Dado and Allen [37], and Paudyal et al. [38], who observed that rumination occurred mostly at night and showed that rumination was highly influenced by activity behavior. Rumination takes place mostly at night when little eating and grazing occur, it is physiologically linked to rest and occurs when the ruminant is relaxed [39]. In contrast, activity behavior, which includes locomotion, standing, and walking, peaked during the afternoon period. This agrees with the finding by Reith et al. [40], who reported that cattle showed the highest activity and lowest rumination during the photophase period. The diurnality index in the present study demonstrated that activity behavior and rumination in beef calves mostly occurred during the scotophase and photophase period of the light–dark cycle, respectively. The result of the present study showed bimodality in the circadian activity behavior of beef calves with two peak values found during the photophase period of the light–dark cycle. This is consistent with the findings by Roelofs et al. [41] and Reith et al. [40] who observed the circadian rhythm of activity behavior in dairy cattle was bimodal with two peak phases occurring during the photophase period.

Activity behavior in beef calves in the present study was found to be at its lowest during the scotophase period and early morning hours, which coincides with the period when the calves spent more time ruminating. Thus, rumination and activity behaviors had distinct inverse diurnal patterns in the present study. The relationship between rumination and activity behavior in the present study was further corroborated by the negative and highly significant correlation coefficient of the former and the latter. The opposite patterns for activity behavior and rumination indicated that beef calves were not able to ruminate and be active simultaneously. These findings are in line with previous studies [42,43,44,45,46,47,48], which reported that rumination showed a clearly diurnal pattern with intensive rumination bouts/frequency occurring during the night and increased activity behavior during the day in the cattle. The finding of the present study agrees with Nikkah et al. [49], who observed that the evolution of ruminant animals has led to specialized behaviors that mainly involve overnight rumination and primary daytime (sunrise and sunset) grazing and eating. Consequently, activity behaviors, such as eating and rumination, have developed unique diurnal and nocturnal rhythms. Marchesini et al. [15] observed that the activity behavior and rumination patterns in young Charolais steers are described not only by how much time is spent on these activities, but also by how these activities are distributed during the course of the day.

The circadian pattern of rumination in this study was similar to what was previously described by Schirmann et al. [46] and Deswysen et al. [50] for heifers; both studies showed that cattle spend more time ruminating at night than during the day. Similarly, the result of the present study revealed a circadian rhythm of rumination as described previously by Krause et al. [51] and Adin et al. [16]. The circadian pattern of activity behavior agrees with the findings by Piccione et al. [30] and Giannetto et al. [52], who observed that cattle exhibited greater activity during the photophase than during the scotophase. Sheanhan et al. [53] opined that activity behavior, such as grazing, is influenced by changes in photoperiod and rumination, and activity behaviors (such as grazing) tend to be inversely correlated. Schirmann et al. [46] observed that rumination was also associated with the time that cattle spent lying down; the relationships between these measures are far from perfect, in part because cows lie down without ruminating and in part because some rumination occurs while cows are standing.

The results of the present study showed no difference in the effect of the breed on the beef calves. This agrees with the results by Stone et al. [54], who reported that rumination times in the Holstein, crossbreed, and Jersey breed of dairy cattle were not significantly different. The results of the present study are inconsistent with the findings by Carvalho et al. [55], who reported that Holstein steers spent more time ruminating than Angus steers. Welch et al. [17] reported that Guernsey cattle had lower rumination times than the Jersey and Holstein breeds of cattle. Both cattle breeds were fed similar diet types and quantities and visual observation was used in the recording of rumination. Differences observed in the present study and the other studies may be due to discrepancies between automatic rumination systems and visual observations. An automated rumination monitoring system can provide a reasonably accurate measurement of rumination in cattle, while traditional methods of measuring rumination by direct observation are laborious and time-consuming [56,57]. In the current study, sex did not affect the duration of rumination in beef calves. This agrees with the findings by Vargas Junior et al. [58] and de Souza Teixeira et al. [59], who reported that sex does not affect rumination activity in Nellore and beef calves.

In the present study, beef calves backgrounded on cover crops had higher rumination than those on perennial pasture. The differences in rumination times may be attributed to variations in the physical structures of the feeds used in backgrounding. This agrees with the findings by Beauchemin [2], Teimouri et al. [60], Yang and Beauchemin [61,62], Nørgaard et al. [63], and Braun et al. [64], who demonstrated that the physical structures or characteristics of the diets affect rumination in cattle. Similarly, previous work has shown that a change in diet, particularly in fibrous plant material [16] and the size of the forage particle [51], can affect the time spent ruminating.

## 5. Conclusions

In conclusion, the results demonstrated that daily variations in activity behaviors and rumination in beef calves followed similar trends during the study period and that the backgrounding diet influenced rumination and activity patterns of Angus and Angus-cross beef calves. However, sex and breed did not influence the rumination and activity behavior of Angus and Angus-cross beef calves. Monitoring rumination behavior and activity with advanced technologies may also provide important information regarding the temporal effects of management strategies on cattle health and welfare. Because our results suggest that the time of day is strongly associated with rumination behavior and activity, interpretation of the duration of cattle rumination and activity should be made with reference to the time of day when behavioral observations are recorded.

## Figures and Tables

**Table 1:** Nutrient composition is the backgrounding diet.

Item	CC		DL	PP
	Tops	Bulbs		
**Nutrient Composition ^1^**				
Moisture	89.62	93.72	41.24	80.33
Dry matter	10.38	6.28	58.76	19.67
NEm, Mcal/kg	1.46	1.67	1.62	1.52
Neg, Mcal/kg	0.87	1.06	1.06	0.93
Starch	-	-	31.43	-
NDF	35.41	20.73	33.35	47.67
CP	21.27	14.77	12.59	21.30
Fat	3.63	1.65	4.25	3.57
Ca	2.03	0.63	0.58	0.58
K	4.4	3.8	1.83	2.56
Mg	0.22	0.17	0.19	0.18
S	0.51	0.45	0.14	0.21

^1^ During backgrounding, animals were allocated to one of three treatments; DL (calves were fed a haylage ration in drylot), PP (calves grazing perennial pastures), or CC (calves grazing summer grown cover crops) for 55 d. Calves received free-choice minerals (Wind & Rain, Purina Animal Nutrition LLC, MN) during the backgrounding phase. Nutrient analysis conducted on weekly feed samples.

**Table 2:** Effects of sex, breed, and backgrounding on rumination and activity in beef calves.

	Rumination (min)	Activity (min)
**Sex**		
Heifer	44.21 ± 2.01	23.79 ± 0.88
Steer	44.44 ± 2.35	23.79 ± 1.01
**Breed**		
Angus-cross	44.31 ± 2.16	24.02 ± 1.02
Angus	44.64 ± 2.18	23.85 ± 0.92
**Backgrounding diet**		
Cover crop	45.33 ± 1.57 *	23.78 ± 0.68
Perennial pasture	43.96 ± 1.47 *	23.49 ± 0.72 *
Dry lot	44.45 ± 1.59	24.16 ± 0.68 *
Overall mean	44.41 ± 2.18	23.86 ± 0.96

Values are expressed as mean ± SEM. * Means within columns having asterisks are significantly (*p* < 0.05) different (n = 62).

**Table 3:** Relationship between rumination and activity behavior in beef calves during the study period.

		Coefficient of Correlation		
	Correlated Parameters	*r*	*p* Value	R^2^
Heifer	Rumination and activity	−0.8517	<0.0001	0.7253
Steer	Rumination and activity	−0.8188	<0.0001	0.6704
Angus	Rumination and activity	−0.8068	<0.0001	0.6509
Angus-cross	Rumination and activity	−0.8882	<0.0001	0.7889

r = Pearson correlation coefficient, R^2^ = coefficient of determination.

**Table 4:** Relationship between environmental parameters, rumination, and activity behavior in beef calves during the study period.

	Coefficient of Correlation		
Correlated Parameters	*r*	*p* Value	R^2^
Ambient temperature and rumination	−0.8503	<0.0001	0.7230
Relative humidity and rumination	−0.9590	<0.0001	0.9197
Wind velocity and rumination	−0.9622	<0.0001	0.9259
Ambient temperature and activity	0.7395	<0.0001	0.5468
Relative humidity and activity	0.9476	<0.0001	0.8980

R = Pearson correlation coefficient, R^2^ = coefficient of determination.

## Data Availability

Data is contained within the article.
